# A Critical Scoping Review of Pesticide Exposure Biomonitoring Studies in Overhead Cultures

**DOI:** 10.3390/toxics10040170

**Published:** 2022-03-31

**Authors:** Christian Tobias Willenbockel, Julia Prinz, Stefan Dietrich, Philip Marx-Stoelting, Cornelia Weikert, Tewes Tralau, Lars Niemann

**Affiliations:** 1 Department for Pesticide Safety, German Federal Institute for Risk Assessment, Max-Dohrn-Str. 8–10, 10589 Berlin, Germany; christian-tobias.willenbockel@bfr.bund.de (C.T.W.); julia.prinz@bfr.bund.de (J.P.); philip.marx-stoelting@bfr.bund.de (P.M.-S.); tewes.tralau@bfr.bund.de (T.T.); 2 Department for Food Safety, German Federal Institute for Risk Assessment, Max-Dohrn-Str. 8–10, 10589 Berlin, Germany; stefan.dietrich@bfr.bund.de (S.D.); cornelia.weikert@bfr.bund.de (C.W.)

**Keywords:** human biomonitoring, pesticides, exposure, operators, workers, residents, bystanders, tree-grown produce, fruits, vine, systemic exposure

## Abstract

The exposure of operators, workers, residents and bystanders to pesticides is of high potential concern. Yet, reports on pesticide residues in the environment and near treated fields often spark debates if such findings might indicate a health risk. Although the underlying models are considered conservative, there are only limited field data on systemic exposure available. As a first step to improve the situation, we conducted a scoping review of state-of-the-art pesticide exposure biomonitoring studies in operators, workers, residents or bystanders. In contrast to existing reviews, we focused on target cultures of potential high pesticide exposure such as tree-grown produce, vine or hops. The search was conducted in Web of Science, Scopus and PubMed. Out of 17 eligible articles, a total of 11 studies met our search criteria, and 6 of them quantified the systemic exposure of humans. The analysis revealed that exposure was mainly driven by application of pesticides and reentry work, resulting in a higher exposure of operators and workers than of residents and bystanders. In nearly all cases, the systemic exposure was below the relevant toxicological reference values. The studies were subsequently analyzed to identify key criteria for a reliable design of a biomonitoring study on pesticide exposure.

## 1. Introduction

In recent decades, there has been growing public concern about the effects of pesticide use on health and the environment. Consequently, there is an increasing number of epidemiological and toxicological studies addressing potential adverse health effects of pesticides in humans under field conditions in which the reliable measurement of exposure is crucial. Unfortunately, this latter point is often not sufficiently fulfilled, particularly in epidemiological studies. This represents a major weakness and is a frequent cause for scientific dispute and criticism [1,2]. 

Generally, when assessing pesticide exposure in epidemiological studies, several approaches have to be distinguished [3]. The most basic assessment is the use of questionnaires with no temporal correlation with the actual application of the pesticides [1,2]. The application event and resulting exposure are mainly reconstructed via a written procedure or interviews. While procedurally easy to perform and cost effective, this approach, by method, bears the risk of recall bias, unless it is substantiated by further data. This can be data from geographic information systems (GISs) such as information from registries of residents living in close proximity to the fields treated, pesticide use registers or working accounts of operators and workers. Additionally, without additional serum measurements or further substantiation with biomarkers, these approaches are inherently limited as they rely on indirect exposure assessments only. This poses a significant challenge for any further verification and interpretation of the results which, in turn, severely impacts their potential scientific, toxicological or regulatory use. 

A promising route towards improvement in the quality, reliability and practical relevance of epidemiological studies is to combine the collection of health outcomes with human biomonitoring (HBM) data for the assessment of real exposure. HBM methods comprise the analytical measurement of active compounds or specific metabolites. Typically, measurements will be conducted in serum or urine [4,5,6], yet potential matrices range from breast milk through hair and nails to dents. Apart from exposure markers, biological effect markers are also frequently measured. Examples comprise the inhibition of plasma or red blood cell acetylcholinesterase, DNA adduct formation or chromosome anomalies [7]. Depending on the marker, the corresponding measurements allow conclusions regarding exposure to single substances or classes of pesticides. For example, inhibition of plasma or red blood cell acetylcholinesterase will indicate exposure to organophosphorous insecticides as a class rather than a single substance. Crucially, the aim of HBM studies is, however, the quantitation of systemic exposure and not the detection of single exposure events (which might well lie in the past). This so-called “back (or reverse) calculation” currently often relies on physiology-based pharmacological or toxicokinetic models (PBPK/PBTK). The reliability of such calculations increases greatly when kinetic data are available as it is the case, for example, for some pyrethroids [8,9]. The resulting exposure estimates can subsequently be compared to the corresponding pesticides’ reference doses. Depending on the scenario, such data can help to retrospectively quantify exposure and evaluate health policies but might also be helpful when trying to substantiate individual case reports or etiologies [10,11,12,13,14]. The combination of HBM and epidemiological tools will certainly increase the reliability of results but is also clearly more demanding in terms of effort, time and costs [3]. Proper assessment of systemic exposure imminently requires that two study criteria be met. First, the biomonitoring sampling has to be timed to the pesticide application because many pesticides, in fact, have a half-life which actually is only in the range of hours [15,16]. Second, repeated sampling is necessary to warrant capturing the respective toxicokinetics [15].

In the present review, we therefore focused on biomonitoring studies which reliably assessed pesticide exposure by means of biomonitoring and, preferably, also quantified the systemic exposure. The latter requirement makes sure that the routes of exposure monitored comprised all three options, that is: (i) inhalative, (ii) oral and (iii) dermal uptake [17]. This is a major advantage as the extent of exposure by each route will obviously differ for residents as compared to operators or workers. Moreover, contrary to previous reviews by Dereumeaux et al. and Teysseire et al. [3,17,18], the focus of the present analysis is on biomonitoring not as an exploratory tool but as a regulatory tool. This includes addressing, for example, the question of observance of reference values.

Two studies were identified as preliminary examples for extensive biomonitoring of pesticide exposure, one being from the Netherlands [16], and the other from the UK [10,19]. Both studies used urine as the primary matrix for the biomonitoring. Apart from the sampling of urine being a non-invasive method, it has the advantage of the respective metabolite measurements being integrative across the various exposure pathways.

From a regulatory point of view, application techniques leading to a potentially high exposure to pesticides are of special concern. Such high (and thus conservative) exposure scenarios are expected particularly for overhead cultures with pesticide application often being performed either by machine-drawn air blast or as a hand-held overhead spray. Examples of such cultures are vine, tree-grown produce, such as apples, or hops. All of these are thus a good starting point for critically assessing pesticide exposure in the field and to draw conclusions for future studies. The further literature search hence was focused on vine, apples and hops with particular attention on studies that used urine or blood as integrative matrices for biomonitoring.

## 2. Materials and Methods

### 2.1. PRISMA Extension

Our scoping review was performed according to the PRISMA extension for scoping reviews (PRISMA-ScR) and the accompanying checklist as proposed by Tricco et al. [20]. In Table S1 of this review, we have added an overview providing information on which pages the various “reporting items” from the checklist have been addressed.

### 2.2. Search Strategy

The literature search was independently performed in the bibliographic databases Web of Science, Scopus and PubMed from 16 to 18 March 2021 by two authors (C.T.W. and J.P.). The construction of the search string followed our research objective and is a specialization of the string of Teysseire et al. [17]. The search string met the demands of our particular research question with its focus on biomonitoring studies with agricultural pesticide application in selected target cultures of potential high exposure (tree-grown produce such as apples, vine or hops). For exposure analysis, we included all groups possibly exposed to pesticides used in agricultural applications in order to assess the safety of pesticides in a regulatory and public health context. These comprised (i) operators, who apply pesticides to plants, (ii) workers, who reenter the crops after pesticide application, (iii) residents, who live nearby fields where pesticides are applied, and (iv) bystanders, who might be exposed accidentally. The inclusion of either urine or serum was mandatory. The search was restricted to articles in English. The construction of the respective search string followed the PICOS principles (participants, interventions, comparisons, outcomes and study design) [3] and read “(proximity or neighborhood) AND (field* OR crop* OR agriculture*) AND (agricultural* OR rural) AND (communit* OR area*) AND (oper* OR operator OR bystander OR appli* OR farm* OR work* OR worker OR workers OR residen* OR resident) AND (urin*OR blood*) AND ((pesticide* OR fungicide* OR herbicide* OR insecticide* OR agrochemical*) AND exposure*) AND (hop OR hops* OR fruit* OR wine* OR vine* OR “tree fruit” OR bushberr* OR orchard* OR pome* OR pomi* OR apple* OR cultivation)”.

In this string, the participants are the operators, workers, residents and bystanders. Intervention is the application of pesticides to crops. It should be noted that this comparison has several dimensions: comparison of the same individuals before and after the spraying event, comparison between different individuals and groups and comparison to reference values. The outcome is the (measured or estimated) exposure to pesticides with the target cultures being part of the study design. 

### 2.3. Data Collection and Storage

The search of all databases led to 86 non-duplicate articles. In total, 108 articles were obtained and regarded as potentially relevant. All articles were stored in an Endnote file which was shared amongst the authors together with an Excel file listing the respective bibliographies and abstracts of all studies.

### 2.4. Selection of Eligible Studies

The eligibility criteria for the selection of studies were based on analyses of existing extensive biomonitoring studies such as Figueiredo et al. [21], Vermeulen et al. [16] and Galea et al. [10,19,22,23]. In short, studies deficient in one or more of the following criteria were excluded:

- No biomonitoring for the assessment of pesticide exposure but sole reliance on a geographic information system, self-reporting or a questionnaire.
- Neither blood nor urine used as a matrix for biomonitoring.
- Non-human studies or methodological studies.
- Reviews.
- Non-observational studies.
- No relationship to pesticide exposure.
- Target cultures other than fruit growing, hops or vine (“wrong target culture”).
- Focus on pesticides banned in Germany for more than six years (since 1 January 2015), e.g., chlorpyrifos.
- Measurement of unspecific biomarkers which only mark the presence of a group of pesticides such as dimethylthiophosphate (DMTP) or dialkylphosphate (DAP) for organophosphates. Such unspecific biomarkers were only considered in those few cases where the study design allowed for clear exposure correlations: for example, if only one pesticide was applied on the target crops during the study.

We did not exclude studies which investigated only one group of operators, workers, residents or bystanders. These groups often overlap, and we supposed that information gained out of the investigation of either one of these groups might be relevant for the others. 

Assessment was independently performed by four authors (C.T.W., J.P., S.D. and L.N.) by screening titles and abstracts and applying the aforementioned exclusion criteria. Individual assessments were then subjected to within-group discussion. Subsequently, C.T.W. and J.P. conducted a full-text analysis of the selected studies and extracted detailed information on study design, characteristics and results. 

## 3. Results

### 3.1. Basic Information

**Figure 1:**
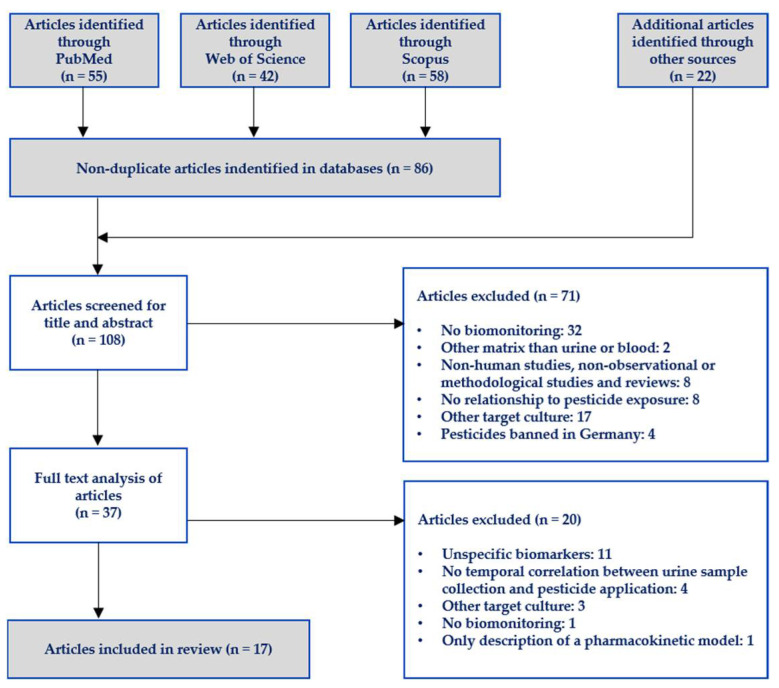
Flowchart of search strategy and study selection with exclusion criteria (based on [20]).

The literature search was performed as depicted in Figure 1. A systematic screen of PubMed, Web of Science and Scopus yielded a total of 155 articles, 86 of which were non-duplicates. Another 22 articles of potential interest were identified by screening their reference lists or full texts and, therefore, were considered to have been obtained “from other sources”.

In a stepwise approach, subsequent analysis of titles/abstracts and full texts filtered out a total of 11 studies published in 17 articles which were eventually included in the review (see Table 1, Table 2, Table 3 and Table 4). It should be noted that articles referring to the same study cohort (as in participants) are considered as belonging to the same study. This is, for example, the case for Kennedy et al. [24] and Fustinoni et al. [25].

#### 3.1.1. Number of Articles Arranged by Target Cultures

We identified nine articles and six studies with vine as the target culture (Table 2) [24,25,26,27,28,29,30,31,32]. For tree-grown produce, we found eight articles based on five studies (Table 1) [10,19,23,33,34,35,36,37], two of which referred to apples [35,36,37]. No reports were found for hops as the target culture. Study data were collected in three regions, namely, North America (three studies [32,35,36,37]), Europe (seven studies [10,19,22,23,24,25,26,27,28,29,30,31,34]) and China (one study [33]), with six of these studies confined to a single region only [24,25,26,27,32,33,36,37].

**Table 1:** Study characteristics of studies on fruit trees as target cultures.

Study	Participants, Sample Size, Location, Year	Culture	Pesticides (Type), Analyzed Metabolites/Pesticides Application Type	Biomonitoring Matrices and Other Samples	Biomonitoring Strategy	Systemic Exposure
Fenske et al., 2003 [36]	**Reentry workers** (apple thinners) (*n* = 20) Three orchards in Washington, DC, USA Year: 1994	Apples (fruit trees)	**Pesticide:** azinphos-methyl (insecticide) **Analyzed metabolites:** dimethylphosphate (DMP), dimethylthiophosphate (DMTP), dimethyldithiophosphate (DMDTP) (specific due to study design) **Application type:** spray	Urine	**Sampling timed to pesticide application:** yes **Background and repeated measurements:** no **24 h urine analyzed:** no **Strategy for urine measurements:** urine spot sample was assumed to represent steady state **Conversion factors determined:** no **Dermal exposure assessed:** no **Environmental measurements:** yes (collection of foliar samples of leaves, see Simcox et al., 1999 [37]) **Study duration:** six-week thinning season **Control group:** no **Personal protective equipment considered:** yes (Simcox et al., 1999 [37]) **Questionnaire/Confounders/Medical pre-examinations considered:** no	**Systemic exposure assessed:** yes, based on known conversion factors and assumptions of urine volume **Conversion factors used:** yes **Correlation between urine and dermal measurements:** no
Simcox et al., 1999 [37]	See Fenske et al., 2003 [36]	See Fenske et al., 2003 [36]	See Fenske et al., 2003 [36]	Urine, serum	**Sampling timed to pesticide application:** yes **Background and repeated measurements:** yes **24 h urine analyzed:** no **Strategy for urine measurements:** spot urine samples were collected daily at the end of the shift for the duration of the whole study, and collection continued one week after end of thinning season (reentry work) **Conversion factors determined:** no **Dermal exposure assessed:** no **Environmental measurements:** no **Study duration:** six-week thinning season **Control group:** no **Personal protective equipment considered:** no (long pants, long-sleeved shirts, cap, work boots or tennis shoes) **Questionnaire/Confounders:** no	Assessed in Fenske et al., 2003 [36]
Galea et al., 2011 [23]	**Residents** living within 100 m of the edge of a field (*n* ≥ 130 adults and 65 children) East Lothian, Kent and Norfolk (UK) Years: 2011 and 2012	Fruit trees, arable crop	**Pesticides:** captan (fungicide), chlormequat (growth regulator), chlorpyrifos (insecticide), cypermethrin (insecticide), deltamethrin (insecticide), diquat (herbicide), iprodione (fungicide), penconazole (fungicide), pirimicarb (insecticide), thiophanate-methyl (fungicide) Analyzed metabolites/pesticides *: not specified **Application type:** spray	Urine	**Sampling timed to pesticide application:** yes **Background and repeated measurements:** yes **24 h urine analyzed:** yes **Strategy for urine measurements:** urine samples collected within two days after spray event; background samples collected within and outside the spray season **Conversion factors determined:** no **Dermal exposure assessed:** no **Environmental measurements:** no **Study duration:** two years **Control group:** no **Personal protective equipment considered:** no **Questionnaire/Confounders/Medical pre-examinations considered:** yes	**Systemic exposure assessed:** no (for systemic exposure, see Galea et al., 2015 [10]) **Conversion factors used:** no **Correlation between urine and dermal measurements:** no
Galea et al., 2015 [19]	See Galea et al., 2011 [23]	Fruit trees, arable crop	**Pesticides:** captan (fungicide), chlormequat (growth regulator), chlorpyrifos (insecticide), cypermethrin (insecticide) Analyzed metabolites/pesticides *: cis-1,2,3,6- tetrahydrophthalimide (THPI), chlormequat *, 3,5,6-trichlorpyridinol (TCP), cis- and trans- 2,2-dichlorovinyl-3,3- dimethylcyclopropane-1-carboxylic acid (DCVA) (specific) **Application type:** spray	Urine	See Galea et al., 2011 [23]	**Systemic exposure assessed:** no (for systemic exposure, see Galea et al., 2015 [10]) **Conversion factors used:** no (for conversion of urinary concentrations, a pharmacokinetic model was applied) **Correlation between urine and dermal measurements:** no
Galea et al., 2015 [10]	See Galea et al., 2011 [23]	Fruit trees, arable crop	See Galea et al., 2015 [19]	Urine	See Galea et al., 2011 [23]	**Systemic exposure assessed:** yes, systemic exposure estimated via regulatory exposure models (Europoem or REA) due to spray information (amount of pesticide sprayed, etc.). **Conversion factors used:** no (for conversion of urinary concentrations, a pharmacokinetic model was applied) **Correlation between urine and dermal measurements:** no
Hines et al., 2008 [35]	Private **operators** (*n* = 74 (73 men, 1 woman)) Iowa, NC, USA Years: 2002 and 2003	Fruit trees (apples and/or peaches)	**Pesticides:** captan (fungicide), thiophanate-methyl (fungicide), benomyl (fungicide) **Analyzed metabolite:** cis-1,2,3,6-tetrahydrophthalimide (THPI) (specific) **Application type:** air blast, hand spray	Urine, dermal exposure, air	**Sampling timed to pesticide application:** yes **Background and repeated measurements:** yes **24 h urine analyzed:** yes **Strategy for urine measurements:** first morning urine one day before pesticide application and 24 h urine after application **Conversion factors determined:** no **Dermal exposure assessed:** yes, via patches at ten different spots on clothes or skin **Environmental measurements:** air within breathing zone **Study duration:** two days for each participant (at least seven days apart) **Control group:** no **Personal protective equipment considered:** yes (input for the AHS algorithm) **Questionnaire/Confounders considered:** yes	**Systemic exposure assessed:** no **Conversion factors used:** no **Correlation between urine and dermal measurements:** yes (significant)
Sams et al., 2016 [34]	**Residents** living within 100 m of the edge of a field (*n* = 48 adults and 6 children) Kent (UK) Years: 2011 and 2012	Fruit trees	**Pesticide:** penconazole (fungicide) **Analyzed metabolites:** penconazole-OH, penconazole-COOH (specific) **Application type:** spray	Urine	See Galea et al., 2011 [23] **Conversion factors determined:** yes, based on a volunteer study (single oral dose of penconazole at the ADI)	**Systemic exposure assessed:** no **Conversion factors used:** yes, determined within a volunteer study **Correlation between urine and dermal measurements:** no
Tao et al., 2019 [33]	**Residents/operators** (*n* = 119), operators’ family members (*n* = 156), urban **control group** (*n* = 42) **Children** (rural children (*n* = 247), urban **control group** (*n* = 53)) Henan Province, China Year: 2017	Fruit trees	**Pesticide:** imidacloprid (IMI) (insecticide) Analyzed metabolites/pesticides *: 6-chloronicotinic acid (6-CNA) and imidacloprid * resulting in ∑IMI (specific due to study design) **Application type:** spray	Urine	**Sampling timed to pesticide application:** yes **Background and repeated measurements:** yes **24 h urine analyzed:** no **Strategy for urine measurements:** six spot urine samples per participant (first morning urine 1 d before spray event and 1 d, 2 d, 3 d, 5 d and 7 d after spray event); one first morning urine sample from each control **Conversion factors determined:** no **Dermal exposure assessed:** no **Environmental measurements:** no **Study duration:** March to June 2017 **Control group:** yes **Personal protective equipment considered:** yes **Questionnaire/Confounders considered:** yes	**Systemic exposure assessed:** no **Conversion factors used:** no **Correlation between urine and dermal measurements:** no

**Table 2:** Study characteristics of studies on vine as the target culture.

Study	Participants, Sample Size, Location, Year	Culture	Pesticides (type), Analyzed Metabolites/Pesticides Application Type	Biomonitoring Matrices and Other Samples	Biomonitoring Strategy	Systemic Exposure
Fustinoni et al., 2014 [25]	**Operators/reentry workers** (*n* = 7) Piedemont, Monferrato region, Italy Year: 2011	Vine	**Pesticide:** tebuconazole (TEB) (fungicide) **Analyzed metabolites:** TEB-OH, TEB-COOH (specific) **Application type:** tractor-mounted air blast or spraying upward with hand-held application equipment	Urine, dermal exposure	**Sampling timed to pesticide application:** yes **Background and repeated measurements:** yes **24 h urine analyzed:** yes **Strategy for urine measurements:** workers collected urine 24 h before application and mostly 48 h after last shift **Conversion factors determined:** no **Dermal exposure assessed:** yes, hand exposure was assessed by collecting hand washing liquids during work for 24 h after exposure **Environmental measurements:** no **Study duration:** 12 working days **Control group:** no **Personal protective equipment considered:** yes **Questionnaire/Confounders considered:** yes	**Systemic exposure assessed:** no (for systemic exposure, see Kennedy et al., 2015 [24]) **Conversion factors used:** no **Correlation between urine and dermal measurements:** yes (significant)
Kennedy et al., 2015 [24]	See Fustinoni et al., 2014 [25]	Vine	See Fustinoni et al., 2014 [25]	See Fustinoni et al., 2014 [25]	See Fustinoni et al., 2014 [25]	**Systemic exposure assessed:** yes, assessed based on measurements of actual dermal exposure in Fustinoni et al., 2014 [25] **Conversion factors used:** no **Correlation between urine and dermal measurements:** yes (significant) (see also results from Fustinoni et al., 2014 [25])
Lopez-Galvez et al., 2020 [32]	Male migrant **reentry workers** (*n* = 20) (neither involved in applying nor mixing pesticides) Sonora, Mexico Year: 2016	Vine	**Pesticides:** imidacloprid (insecticide), clothianidin (insecticide), thiamethoxam (insecticide), acetamiprid (insecticide), thiacloprid (insecticide) Analyzed metabolites/pesticides *: 5-hydroxy-imidacloprid (5-OH-IMI), imidacloprid *, clothianidin *, acetamiprid-N-desmethyl, acetamiprid *, thiacloprid * (specific) **Application type:** drip irrigation	Urine, dermal exposure, air	**Sampling timed to pesticide application:** yes **Background and repeated measurements:** no **24 h urine analyzed:** no **Strategy for urine measurements:** one urine sample per participant (first morning urine five days after pesticide application) **Conversion factors determined:** no **Dermal exposure assessed:** yes, assessed using hand wipes **Environmental measurements:** yes, air measurements within breathing zone of workers **Study duration:** winter and summer seasons in 2016 **Control group:** no **Personal protective equipment considered:** yes **Questionnaire/Confounders considered:** yes	**Systemic exposure assessed:** no **Conversion factors used:** no **Correlation between urine and dermal measurements:** yes (significant)
Mandic-Rajcevic et al., 2018 [30]	Male, healthy right-handed **operators** (mixing, application and equipment maintenance work) (*n* = 29) Mantova and Pavia Provinces of the region of Lombardy (Northern Italy) Year: 2011	Vine	**Pesticide:** mancozeb (fungicide) **Analyzed metabolite:** ethylene-bis-thiourea (ETU) **Application type:** closed and filtered tractors (*n* = 29) vs. open tractors (*n* = 9)	Urine, dermal exposure	**Sampling timed to pesticide application:** yes **Background and repeated measurements:** yes **24 h urine analyzed:** yes **Strategy for urine sampling:** 24 h pre-exposure and 24 h post-exposure urine **Conversion factors determined:** no **Dermal exposure assessed:** yes, external pads on clothes for potential exposure measurements and internal pads on skin for actual exposure measurements (modified OECD patch methodology), collection of hand washing liquid (24 h post-exposure) **Environmental measurements:** no **Study duration:** 38 working days in April to July 2011 **Control group:** no **Personal protective equipment considered:** yes, assessment of effect of coveralls and gloves on exposure **Questionnaire/Confounders considered:** yes	**Systemic exposure assessed:** no (systemic exposure and comparison with reference values stated in Mandic-Rajcevic et al., 2019 [29]) **Conversion factors used:** no **Correlation between urine and dermal measurements:** yes (significant)
Mandic-Rajcevic et al., 2019 [29]	See Mandic-Rajcevic et al., 2018 [30]	Vine	See Mandic-Rajcevic et al., 2018 [30]	See Mandic-Rajcevic et al., 2018 [30]	See Mandic-Rajcevic et al., 2018 [30]	**Systemic exposure assessed:** Yes, systemic exposure assessed via patch measurements on clothes (potential exposure) and skin (actual exposure). **New method for data analysis accounting for duration of exposure compared to establish fixed fractional approach which assumes a standard working day of 8 h.** **Conversion factors used:** no **Correlation between urine and dermal measurements:** yes (significant) (see also results from Mandic-Rajcevic et al., 2018 [30])
Mandic-Rajcevic et al., 2020 [31]	Male **operators** (*n* = 16) (subgroup from Mandic-Rajcevic et al., 2018 [30]) Mantova and Pavia Provinces of the region of Lombardy (Northern Italy) Year: 2011	Vine	See Mandic-Rajcevic et al., 2018 [30] However, smaller sample size: closed and filtered tractors (*n* = 11), open tractors (*n* = 5)	See Mandic-Rajcevic et al., 2018 [30]	See Mandic-Rajcevic et al., 2018 [30]	**Systemic exposure assessed:** yes, systemic exposure assessed via patch measurements on clothes (potential exposure) and skin (actual exposure) **Conversion factors used:** no **Correlation between urine and dermal measurements:** yes (significant) (see also results from Mandic-Rajcevic et al., 2018 [30])
Medda et al., 2017 [28]	Chianti (iodine-deficient growing area): Male **workers** (*n* = 29) Male **controls** (*n* = 24) Bolzano: Male **workers** (*n* = 148) Male **controls** (*n* = 40) *n* = 170 workers involved in mixture and application, *n* = 7 workers in reentry work vineyards in Chianti and Bolzano areas, Italy Year: 2017	Vine	**Pesticides:** mancozeb (fungicide) **Analyzed metabolite:** ethylene-bis-thiourea (ETU) (specific for EBDC fungicides and due to study design) **Application type:** no	Urine (ETU and urinary iodine concentration (UIC)), serum (iodine biomarkers for health assessment of thyroid (tyroglobulin (Tg), (free) triiodothyoidine (T3,FT3), free thyroxine (T4, FT4)), thyroid volume assessed by ultrasonography	**Sampling timed to pesticide application:** yes **Background and repeated measurements:** no **24 h urine analyzed:** no **Strategy for urine measurements:** spot urine samples collected the day after the treatment for operators/mixers and one day after the reentry in culture for reentry workers **Conversion factors determined:** no **Dermal exposure assessed:** no **Environmental measurements:** no **Study duration:** June to September 2017 **Control group:** yes **Personal protective equipment considered:** yes **Questionnaire/Confounders/Medical pre-examinations considered:** yes **Other:** blood samples for serum analysis for thyroid health effects collected after six weeks from the last treatment in October (time of grape harvest)	**Systemic exposure assessed:** no **Conversion factors used:** no **Correlation between urine and dermal measurements:** no
Mercadante et al., 2019 [27]	**Operators/reentry workers** (*n* = 22), different regions Lombardy, Italy Year: 2012	Vine	**Pesticide:** penconazole (fungicide) **Analyzed metabolites:** PEN-OH, PEN-COOH (specific) **Application type:** sideways spraying tractor-mounted air blast or spraying upwards with hand-held application equipment	Urine, dermal exposure	**Sampling timed to pesticide application:** yes **Background and repeated measurements:** yes **24 h urine analyzed:** yes **Strategy for urine measurements:** 24 h before and 48 h after last shift, 42 mixing and applications and 12 reentries monitored **Conversion factors determined:** no **Dermal exposure assessed:** yes, assessment of potential and actual body exposure via pads **Environmental measurements:** no **Study duration:** May to July 2012 **Control group:** no **Personal protective equipment considered:** yes **Questionnaire/Confounders considered:** yes	**Systemic exposure assessed:** no **Conversion factors used:** no **Correlation between urine and dermal measurements:** yes (significant)
Sleeuwenhoek et al., 2007 [26]	**Operators** (*n* = 8), **reentry workers** (*n* = 1), **bystanders** (*n* = 7) UK Year: 2004	vine (potatoes)	**Pesticide:** mancozeb (fungicide), cypermethrin (for potatoes)^+^ (insecticide) **Analyzed metabolite:** ethylenethiourea (ETU) (specific for EBDTC fungicides) **Application type:** hand-held, air-assisted, boom	Urine	**Sampling timed to pesticide application:** yes **Background and repeated measurements:** no **24 h urine analyzed:** no **Strategy for urine measurements:** one urine sample per participant (first morning urine after last exposure of the week) **Conversion factors determined:** no **Dermal exposure assessed:** yes, estimated based on EUROPOEM database and the regulatory risk assessment process **Environmental measurements:** no **Study duration:** one year **Control group:** no **Personal protective equipment considered:** yes **Questionnaire/Confounders/Medical pre-examinations considered:** yes **Other:** inhalative and oral exposure routes were assumed to be negligible	**Systemic exposure assessed:** yes, systemic exposure estimated via regulatory exposure models (EUROPOEM and REA) due to spray information (amount of pesticide sprayed, etc.) **Conversion factors used:** no **Correlation between urine and dermal measurements:** no (but estimates for dermal exposure used to predict urinary concentrations)

**Table 3:** Results of studies on fruit trees as target cultures.

Study	Exposure Measurements	Conclusion/Results	Critique
Fenske et al., 2003 [36]	**Units of urine measurements:** µmol/L **Comparison to reference values:** reference values mostly observed; 2.4% of doses exceeded California EPA reference value for reentry > 14 days (geometric mean of 19 µg/kg and day), but 27% of doses exceeded reference value for reentry < 14 days (geometric mean 42 µg/kg and day). **Results of personal protective equipment:** PPE not considered	**Results of exposure:** Exposure after application higher than before when measured in µg/L urinary excretion and dermal exposure. TEB-OH metabolite peaked within 24 h after application. None of the doses exceeded US EPA guidance value of 560 µg/kg and day, but in 2.4 (reentry > 14 days) to 27% (reentry < 14 days) of all cases, there was transgression for reference value of CAL US EPA of 76 µg/kg and day. Comparison of reentry periods of more or less than 14 days. Bioconversion factors for azinphos-methyl applied for estimation of systemic exposure. Exposure was found to depend on reentry timing. Exposure was lower for reentry after more than 14 days. **Health-related outcomes:** not assessed	No determination of background concentrations prior to pesticide application (important parameter for the study design). No repeated measurements subsequent to exposure. Investigation of only a single sample. Systemic exposure was assessed by assuming the excreted urine volume since the beginning of the pesticide exposure. No residents and operators considered (only reentry workers). No control group. Only azinphos-methyl investigated.
Simcox et al., 1999 [37]	See Fenske et al., 2003 [36]	See Fenske et al., 2003 [36] **Health-related outcomes:** cholinesterase (ChE) activity monitored, no adverse outcomes noticed	See Fenske et al., 2003 [36]
Galea et al., 2011 [23]	Results presented in Galea et al., 2015a [19], Galea et al., 2015b [10] and Sams et al., 2016 [34]	Description of study protocol. Results are presented in Galea et al., 2015a [19], Galea et al., 2015b [10], Galea et al., 2017 [22] and Sams et al., 2016 [34]. **Health-related outcomes:** not assessed	See Galea et al., 2015 [19] and Galea et al., 2015 [10]
Galea et al., 2015 [19]	**Units of urine measurements:** µg/L and µg/g creatinine **Comparison to reference values:** no **Results of personal protective equipment:** PPE not considered	Very low biomarker concentrations for captan and cypermethrin were found (approximately 90% of the samples < LOD). **Results of exposure: For chlorpyrifos and chlormequat, no significant in-** **crease in urinary biomarker concentrations upon spray event were observed.** Urinary biomarker concentrations for chlormequat were found to be higher within compared to outside the spray season. **Health-related outcomes:** not assessed	Systemic exposure not assessed. Only residents considered, no operators or reentry workers. No control group. No comparison to reference values.
Galea et al., 2015 [10]	**Units of urine measurements:** µg/L and µg/g creatinine **Comparison to reference values:** yes, AOEL was not reached for any of the pesticides **Results of personal protective equipment:** PPE not considered Blindly estimated urinary excretion of metabolites was compared to actual measurements from Galea et al., 2015a [19] and Sams et al., 2016 [34]	**Results of exposure: Very low effect of spray event on the overall pesticide exposure of residents.** Predictive values based on the REA-PK model were found to be sufficiently conservative. **Health-related outcomes:** not assessed	Systemic exposure only indirectly assessed via exposure models. No assessment of the systemic exposure on the basis of urine measurements and conversion factors. Only residents considered, no operators or reentry workers. No control group.
Hines et al., 2008 [35]	**Units of urine measurements:** µg/L and µg/g creatinine, excretion rates in µg/h **Comparison to reference values:** no **Results of personal protective equipment:** reduction factor for PPE as input for the AHS algorithm	**Results of exposure:** Generally, captan and THPI were most frequently detected when pesticides were applied via air blast. Highest concentrations of THPI in urine were found on the morning after pesticide application. Significant correlations between internal (urine) and external (air, hand rinse, dermal patches) exposure measurements were found (**strongest correlation between captan concentrations in hand rinse samples and THPI concentrations in urine**). Highest dermal exposure was found on hands, forearms and thighs. AHS algorithm was significantly and marginally predictive of thigh and forearm exposures, but did not predict air, hand rinse or urinary THPI exposures. **Health-related outcomes:** not assessed	**Systemic exposure not assessed.** Since the private application of pesticides was investigated, a proper separation between operators and residents is not possible. No control group. No comparison to reference values.
Sams et al., 2016 [34]	**Units of urine measurements:** µmol/mol creatinine **Comparison to reference values:** yes, highest urinary concentration was approximately 100 x lower than the peak excretion after an oral dose at the ADI **Results of personal protective equipment:** PPE not considered	Penconazole-OH and penconazole-COOH suitable as urinary biomarkers to assess systemic exposure of penconazole. **Results of exposure:** Very low biomarker concentrations for penconazole were found (>80% of the samples < LOD). Results used to estimate systemic exposure of penconazole within a study from Fustinoni et al., 2015 [38]. **Health-related outcomes:** not assessed	**Systemic exposure not assessed.** Only residents living in close proximity to target fields were considered. No operators and/or reentry workers considered. No control group. Human volunteer studies of this type in order to determine conversion factors are not allowed in Germany.
Tao et al., 2019 [33]	**Units of urine measurements:** ng/mL and µg/g creatinine **Comparison to reference values:** no **Results of personal protective equipment:** personal protective measures of operators were found to be insufficient	**Results of exposure:** Target metabolites were found in 100% of the urine samples. Increase in IMI exposure in rural residents after pesticide application, with significantly higher concentrations for operators. **Highest IMI concentrations in rural residents were found 2 d after spray event.** Significant impacts of diet, sex, age and region on exposure to IMI observed. **Health-related outcomes:** not assessed	Systemic exposure not assessed. No comparison to reference values. Not clear whether apple cultivation was considered (only “orchards” in general mentioned).

**Table 4:** Results of studies on vine as the target culture.

Study	Exposure Measurements	Conclusion/Results	Critique
Fustinoni et al., 2014 [25]	**Units of urine measurements:** µg/L and µg/g creatinine, excretion rates in µg/h **Comparison to reference values:** reference values assessed in Kennedy et al., 2015 [24] **Results of personal protective equipment:** Non-uniform PPE and different efficacy might be two of the factors for the observed range of excretion rates for TEB-OH and TEB-COOH. Median protection factor of 98% provided by wearing overalls.	**Results of exposure:** Exposure after application higher than before when measured in µg/L urinary excretion and dermal exposure. TEB-OH metabolite peaked within 24 h after application. Correlation of total dermal exposure measurements and post-application 24 h urinary biomarker measurements of TEB- OH and TEB-COOH was significant, r = 0.756 and r = 0.577. Possibly suitable approach for future studies. Very small number of participants (*n* = 7) noted as major limit. Hands accounted for 17 to 86% of actual skin exposure. **Health-related outcomes:** not assessed	Systemic exposure not stated (see Kennedy et al., 2015 [24]). In Kennedy et al., 2015 [24], systemic exposure only determined via dermal exposure and model calculations, but not directly on the basis of urine measurements. No use of conversion factors. Very small sample size (*n* = 7). No residents. No control group.
Kennedy et al., 2015 [24]	**Units of urine measurements:** see Fustinoni et al., 2014 [25] **Comparison to reference values:** Reference values assessed by dermal exposure and model predictions of ACROPOLIS model. Reference values observed in both cases. Applied model gives good prediction of exposure based on urinary measurements. **Results of personal protective equipment:** higher actual dermal exposure predicted if pesticide is applied without gloves	**Results of exposure:** Used study data from Fustinoni et al., 2014 [25]. Urinary measurements were compared with prediction from exposure model for all sources of exposure (dietary and non-dietary). Systemic exposure was measured based on dermal exposure measurements. Correlation with urinary measurements was assessed. Model predictions of systemic exposure seemed to be reliable, but very small (*n* = 7) sample size. **Health-related outcomes:** not assessed	Systemic exposure only determined via dermal exposure and model calculations, but not directly on the basis of urine measurements. No use of conversion factors. Very small sample size (*n* = 7). No residents. No control group.
Lopez-Galvez et al., 2020 [32]	**Units of urine measurements:** µg/L and µg/g creatinine **Comparison to reference values:** no **Results of personal protective equipment:** training on PPE usage was found to significantly reduce IMI concentrations in hand wipes	**Results of exposure:** Imidacloprid was most frequently detected among all neonicotinoid biomarkers (in 95% of the urine samples). **Strong correlation between imidacloprid concentrations measured in hand wipes and urinary 5-OH-IMI.** Hand wipes stated as a possible alternative to urinary biomonitoring. Concentrations of imidacloprid in air < LOD. Concentrations of urinary 5-OH-IMI significantly higher in summer. **Health-related outcomes:** not assessed	No background or repeated measurements. Systemic exposure not assessed. No control group. No comparison to reference values.
Mandic-Rajcevic et al., 2018 [30]	**Units of urine measurements:** µg/L and µg/g creatinine **Comparison to reference values:** for reference values, see Mandic-Rajcevic et al., 2019 [29] **Results of personal protective equipment:** Coveralls reduced skin exposure by 4 times (open tractors) and 10 times (closed tractors). Gloves led to 10 times lower hand exposure during application with open tractors. Gloves led to an increase in exposure when closed tractors were used, due to suspected transport of contaminated gloves in tractor cabins.	**Results of exposure:** Comparison of individual levels pre- and post-exposure dependent on if the tractor was open or closed during application. ETU level post-exposure significantly higher than pre-exposure (*p* < 0.001). Absolute levels higher in most individual comparisons. Statistically significant positive correlation between total skin exposure and ETU levels (r = 0.55, *p* < 0.001). Dermal exposure contributed more than 90% of total skin dose. ETU is a suitable biomarker for occupational exposure to mancozeb, but urinary measurement values cannot be assessed directly because of a lack of biological exposure limits. **Further studies concerning use of correlation between total skin exposure and urine biomonitoring advocated.** **Health-related outcomes:** not assessed	Systemic exposure determined via dermal measurements. In a second step, comparison/correlation with urine measurements. No direct assessment of the systemic exposure on the basis of urine measurements and conversion factors. Small sample size (*n* = 29), only operators (only men). No residents. No control group.
Mandic-Rajcevic et al., 2019 [29]	**Units of urine measurements:** µg/L and µg/g creatinine **Comparison to reference values:** yes, even less conservative methods give estimate of several hundred times below AOEL reference value of 0.02 mg/kg bw **Results of personal protective equipment:** see Mandic-Rajcevic et al., 2018 [30]	**Results of exposure:** Median pre-exposure 24 h ETU urine 0.93 and 0.51 µg/g creatine for open and closed tractors. Median post-exposure 24 h ETU urine 1.83 and 1.22 µg/g creatine for open and closed tractors. Use of new calculation method for calculation of systemic exposure accounting for duration of exposure yields a reduced dose of 50%, 81% and 80% for body, hands and total absorbed dose when compared to established fixed fractional approach. Systemic exposure assessment dependent on chosen approach. New model yielded better correlation of dermal pad methodology for total body dose and urine measurements post-exposure. It was noted that hand exposure contributed 97 % to total skin exposure but the correlation with post-exposure urine measurements of free ETU for dermal hand exposure (ρ = 0.41) (time-adjusted) was lower than for the dermal body exposure (ρ = 0.58) (fixed time) and total dose (ρ = 0.51) (fixed time). Correlation of hand exposure and free ETU urine levels improved by duration-adjusted method. **Health protection recommendations:** regular hand washing might considerably lower the absorbed dose via skin	See Mandic-Rajcevic et al., 2018 [30].
Mandic-Rajcevic et al., 2020 [31]	**Units of urine measurements:** µg/L **Comparison to reference values:** yes, exposure of highest exposed operator was 1000 times lower compared to the AOEL of 0.035 mg/kg for mancozeb **Results of personal protective equipment:** see Mandic-Rajcevic et al., 2018 [30]	**Results of exposure:** Mean absorbed dose (dermal exposure) was 0.9 ng/kg (from body exposure: 0.1 ng/kg; from hand exposure: 0.6 ng/kg). Estimation of EBEL (equivalent biologic exposure value) for mancozeb by combining the results for dermal exposure (see Mandic-Rajceciv et al., 2018 [30]) and consideration of the AOEL for mancozeb as guidance value. Approach resulted in an EBEL of 0.15 mg (free ETU in urine) and 0.7 mg (total ETU in urine) for mancozeb. Exposure of the highest exposed operator was at the level of 20% of the EBEL; the values for all the other operators were less than or equal to 3% of the EBEL. In future, the concept of EBEL might be applied as a screening method in order to estimate the risk of pesticide operators. **Health-related outcomes:** not assessed	See Mandic-Rajcevic et al., 2018 [30]
Medda et al., 2017 [28]	**Units of urine measurements:** µg/L **Comparison to reference values:** no **Results of personal protective equipment:** frequency of use of personal protective equipment in Bolzano workers higher (97.2%) than in Chianti workers (85.2%) (significant, *p* = 0.01)	Mild thyroid-disrupting effect due to mancozeb exposure. Higher thyroid health effects in workers in areas with an iodine deficit (Chianti) than in areas (Bolzano) with an established program for iodine supplementation. **Health-related outcomes:** Lower mean FT4 iodine serum levels in exposed workers in iodine-deficient Chianti area. Increased iodine urinary excretion of >250 µg/L more frequently in higher exposed workers (>20 µg/L ETU) than in less exposed workers. This effect was stronger in Chianti (iodine deficit) than in Bolzano (iodine sufficient) area. Workers in an area with iodine deficit had lower thyroid volumes (≤6 mL) than the respective control groups.	No background or repeated measurements. Systemic exposure not assessed. No comparison to reference values.
Mercadante et al., 2019 [27]	**Units of urine measurements:** µg/L **Comparison to reference values:** no **Results of personal protective equipment:** potential body exposure without clothing and actual body exposure compared, clothing provided good protection (1/100 to 1/1000 lower than potential exposure)	**Results of exposure:** Measurements of exposure ranging from 15.6 to 27.6 µg/L for PEN-OH and 2.5 to 10.2 µg/L for PEN-COOH. Excretion rate of PEN-OH had a peak within 24 h post-exposure. PEN-OH could possibly be used for biomonitoring on a regular basis. Hand exposure of reentry workers was found to be a major factor for overall exposure. Correlation found between total dermal exposure and urinary excretion of metabolites. Concentration of PEN-OH 24 h and 24 to 48 h after work shift correlated with actual body and total dermal exposure (0.279 ≤ r ≤ 0.562). Described a method for assessing if participants provided all urine samples based on creatine levels. **Health-related outcomes:** not assessed	Systemic exposure not assessed. Very small sample size (*n* = 22). No residents. No control group. No comparison to reference values.
Sleeuwenhoek et al., 2007 [26]	**Units of urine measurements:** µg/L and µg/g creatinine **Comparison to reference values:** yes, measured urine biomarker concentrations indicate that the ADI is not reached **Results of personal protective equipment:** no protective equipment assumed for bystanders in modeling	Urinary ETU concentrations were highest for sprayers. Median ETU concentrations for post-application workers and bystanders were < LOD. **Overall, predicted urinary concentrations were higher than the observed ones. Estimated values based on regulatory risk assessment were found to be sufficiently conservative.** Pre-exposures of mancozeb have to be taken into account due to its long half-life of 100 h. Results possibly not representative due to small sample size. **Results of exposure:** Measurements of exposure ranging from 15.6 to 27.6 µg/L for PEN-OH and 2.5 to 10.2 µg/L for PEN-COOH. Excretion rate of PEN-OH had a peak within 24 h post-exposure. The results for cypermethrin are not discussed here, since different target cultures were covered. **Health-related outcomes:** not assessed	No background or repeated measurements. Systemic exposure only estimated by using a model calculation including the amount of pesticides. No direct assessment of the systemic exposure on the basis of urine measurements. No use of conversion factors. Small sample size: 7 bystanders, 8 sprayers, 1 worker. No control group.

#### 3.1.2. Participants and Sample Size

In most studies, the participants were allocated to group cohorts depending on their (professional) role. Generally, the studies distinguished between operators, reentry workers and residents. Operators [24,25,26,27,29,30,31,33,35] and reentry workers [24,25,26,27,28,32,36,37] were considered in six studies and residents in three [10,19,22,23,33,34]. The corresponding cohort sizes varied significantly, with *n* = 7–170, 1–22 and 54–403 for operators, reentry workers and residents, respectively. It should be noted that depending on the study design, there can be overlaps with regard to the various groups. For example, in Mercadante et al. [27], operators also performed reentry work. Additionally, the definition of residents varied. All studies considered residents to be persons living in a certain radius of the treated fields. However, depending on the study, the size of the respective radius would range from 50 m to 250 m. Only one study explicitly included bystanders (*n* = 7) [26], and only two studies had control groups [28,33].

#### 3.1.3. Pesticides Used

As might be expected, pesticide application varied depending on the culture investigated. For tree-grown produce (Table 1), the corresponding active substances covered a range of protective uses and comprised azinphos-methyl (organophosphate insecticide) [36,37], captan (fungicide) [10,19,35], chlormequat (growth regulator) [10,19], chlorpyrifos (insecticide) [10,19], cypermethrin (insecticide) [10,19], deltamethrin (insecticide) [10,19], diquat (herbicide) [10,19], iprodione (fungicide) [10,19], penconazole (fungicide) [10,19,34], pirimicarb (insecticide) [10,19], thiophanate-methyl (fungicide) [10,19,35], benomyl (fungicide) [35] and imidacloprid (insecticide) [33]. Contrastingly, in vine (Table 2), application mainly included fungicides such tebuconazole [24,25], mancozeb [26,28,29,30,31] and penconazole [27]. Lopez-Galvez et al. [32] also investigated the insecticides imidacloprid, clothianidin, thiamethoxam, acetamiprid and thiacloprid.

### 3.2. Sampling Strategies

The study duration ranged from 12 days [28,29] to up to 2 years [10,23,26,27], with all studies relying on urine as the preferred matrix and only two studies additionally sampling serum [19,28]. Measures for urinary metabolites are usually given as concentrations (µg/L, µmol/L, µg/g creatinine or µmol/mol creatinine), with only a few studies also referring to rates (excretion in µg/h [29]). In all cases, care was taken to carefully time sampling to actual substance usage with the participating farmers calling in prior to application. Concomitant measurements of background exposure were performed in seven studies looking at tree-grown produce (*n* = 4, please see Table 1) [10,19,23,33,34,35,37] or vine (*n* = 3, please see Table 2) [24,25,27,30]. Except for Simcox et al. [37], all these studies additionally employed a questionnaire asking the study participants about living conditions and/or possible confounders. Of the remaining studies, three relied on questionnaires only for their background information [26,28,32].

The studies measuring background exposure showed considerable variation with regard to the respective sampling schemes. While Hines et al. [35] and Tao et al. [33] collected the first morning urine on the day before active substance application, Simcox et al. [37] relied on baseline measurements over a period of at least two weeks prior to the first application. In addition, the latter also collected reference samples from the general, non-agricultural population. Contrastingly, Galea et al. and Sams et al. collected up to three samples during the spraying season which were not correlated with an application event, and up to three additional samples outside the spraying season [10,19,23,34].

For the subsequent sampling of actual exposure, the studies either relied on spot sampling or continuous sampling. Simcox et al. [37] collected daily spot urine samples at the end of each work shift. Sampling was commenced for another week after application and occupational exposure in the thinning season. Other studies used less systematic sampling regimes such as Galea et al. and Sams et al., who collected urine samples within two days after the spray event [10,19,23,34], or Tao et al. [33], who relied on spot urine of the first, second, third, fifth and seventh days after the substance application. Meanwhile, Lopez-Galvez et al. [32] collected morning urine on the first five days after application. Comparable sampling strategies were followed by Medda et al. [28], who collected spot urine samples from operators and mixers the day after substance treatment and from reentry workers the day after starting reentry work. Likewise, Sleeuwenhoek et al. [26] collected one sample per participant following the last exposure of the week. In contrast, several other studies [24,25,27,30,35,37] collected the complete 24 h urine after substance application, with some studies extending the sampling period to include the 24 h before [25,27,30] and 48 h thereafter [27,38]. Notably, in the three latter studies, the amount of urinary metabolites correlated best with the respective dermal exposure assessments.

### 3.3. Exposure

#### 3.3.1. Routes of Exposure

There are three major routes of exposure. These are: (i) the inhalative route, (ii) the dermal route and (iii) the oral route. The individual contributions of these routes to the overall exposure will obviously vary for the different cohorts monitored. For example, an operator inhaling vapors will experience a different exposure than a worker coming into contact with contaminated foliage or somebody ingesting dust particles. While urine measurements are integrative, other matrices or environmental measurements might therefore prove to be more appropriate when it comes to further dissecting the particular contributions to overall exposure. Furthermore, absorption and metabolism will strongly depend on the route of uptake, explaining some of the observed differences in metabolite patterns and kinetics [16,35].

#### 3.3.2. Inhalative Exposure

Inhalative exposure can be measured in a non-invasive way by analysis of (breathing) filters or measurements of ambient air in the respective breathing zones [25,35,39,40]. The latter approach is often the method of choice due to ease of use and was thus used in the few studies that looked at the respective contribution of inhalation to overall exposure. For imidacloprid, the inhalative route accounted for less than 1% of the total exposure, with air concentrations being below the detection limit in the breathing zone of the workers [41,42]. Contrastingly, captan was detected in more than 60% and 32.8% of personal breathing zone samples during application by air blast or hand spray, respectively [35]. This is in line with previous studies which also found inhalation to be a major route of exposure for captan [43,44,45,46].

#### 3.3.3. Dermal Exposure

Skin exposure was assessed experimentally in several studies, most of them on vine [24,25]. One study also modeled exposure based on EUROPOEM [26]. For apples, Hines et al. used patches and hand rinses to assess potential substance exposure for operators [35]. In this study, rinse sampling was restricted to only one hand per person to minimize uncertainties due to error propagation. 

Other studies used different approaches. For example, Fustinoni et al. relied on the collection of hand washing liquids in order to distinguish between potential and actual exposure to tebuconazole in vineyards 24 h after application [25]. This sets the amount detected in clothes against what was washed off the skin. Exposure of the head was recorded separately using non-woven fabric head covers. Likewise, Mandic-Rajcevic et al. used a modified OECD patch methodology and the collection of hand washing liquids over 24 h post-exposure for determining actual and potential body skin exposure to mancozeb in vineyards [30], while Mercadante et al. similarly looked at penconazole [27]. Meanwhile, Lopez-Galvez et al. analyzed hand wipes of reentry workers for imidacloprid [32].

Several of the aforementioned studies identified hand exposure as a major contributor to the overall dermal substance load as well as to total exposure (Table 3 and Table 4). Other sites of contact were the forearms and thighs [35]. Depending on the substance, study design and group looked at, hands accounted for 17 to 97% of skin exposure [29,30]. This contribution was particularly large for reentry workers for whom the data also showed the largest variability [27]. Such variability could pose a problem with regard to data evaluation. With the respective range spanning an order of magnitude of 10^4^, the most likely explanation is a varying use of gloves in this group. 

#### 3.3.4. Other Factors Influencing Exposure—Repeated Exposure, Substance Kinetics and Means of Application

Many of the less systematic studies focused on actual substance application as the major exposure event. The logic behind this is that, for groups such as operators and bystanders, application will constitute a good part of the overall load experienced. Indeed, for captan and cypermethrin, residential biomarker concentrations clearly correlated with substance application, with approximately 90% of the samples being below the limit of detection (LOD) outside the season [19]. However, repeated contact can also have a significant impact. This is most clearly seen for azinphos-methyl, where total worker exposure strongly depended on the number of days between application and reentry (Table 3) [36]. When the reentry time was longer than 14 days, only 2.7% of the specimens exceeded the reference value of 76 µg/kg bw and day for CAL EPA, whereas a reentry time of less than 14 days led to 27% of all specimens being above the reference value. For reentry, the respective dose–response relationship therefore strongly depends on the start of the work. Befittingly, Simcox et al. reported generally low urinary baselines before reentry, except for the one cohort that had started work prior to the start of the study [37]. 

In addition, studies need to accommodate individual substance kinetics to obtain reliable measurements. For imidacloprid, there was an 8.5–15-fold increase in metabolite levels in rural residents and operators one day after the spray event, with the residential levels peaking on day two [33]. Moreover, exposure to imidacloprid was found to be ubiquitous for the respective rural population, with metabolites found in 100% of the sampled cohort. Notably, other substances will follow different kinetics. This is exemplified by penconazole, where urinary metabolite excretion peaked 24 h after exposure at work [27].

Finally, the application technique (air blast or hand-held spray) will, of course, influence the exposure route of a particular active substance and should thus be taken into account [35]. 

#### 3.3.5. Use of Personal and Other Protective Equipment

The use of personal protective equipment (PPE) and other protective equipment obviously has a huge impact on potential exposure. It is hence a key parameter when assessing potential health impacts for workers and operators alike. Unsurprisingly, several studies focused on the impact of PPE on substance exposure. For apples, two studies investigated the influence of PPE [35]. Both studies saw clear effects, recommending the inclusion of a reduction factor for pesticide exposure in the applied exposure model (AHS model) as a result. For vine, the varying use of PPE was suspected to be partly responsible for the observed range of the metabolite excretion rates [25]. In particular, the use of work suits and gloves was found to have a significant impact, with the former reducing dermal exposure by up to 98% [25]. Training on the correct use of PPE was found to be another significant factor for exposure minimization [32].

For operators, PPE proved to be similarly effective. In tractors, work suits reduced skin exposure by 4 to 10 times, depending on whether it was an open (OT) or closed and filtered system (CFT) [30]. Interestingly, while the use of gloves led to a 10 times lower exposure during application in OTs, exposure in CFTs increased. The most likely reason for this initial paradoxical result is operators taking their used gloves into the cabins. In terms of CFTs, these are semi-closed systems with any contaminated PPE, hence leading to an increased inhalative load. Likewise, Mercadante et al. found that clothing provided good protection since actual exposure was 100 or even 1000 times lower than the potential exposure [27]. Nevertheless, it should be noted that the use of PPE can vary significantly depending on availability, awareness or work culture. This is exemplified by the results of Medda et al., which found percentages of PPE use amongst workers to differ from 85.2% to 97.2% [28]. Additionally, broad use of PPE is obviously not an option for bystanders. 

### 3.4. Assessment of Systemic Exposure

Biomarker measurements allow conclusions on the overall substance load experienced. However, any further assessment needs information on the systemic exposure as the potentially effective dose. To calculate this, more information is needed such as substance-specific conversion factors and toxicokinetics. Systemic exposure was assessed in several of the reviewed studies [36]. 

Most authors relied on conversion factors to calculate the absorbed dose from the respective (urinary) metabolite measurements. Fenske et al. [36] and Sams et al. [34] used oral conversion factors to assess systemic exposure for azinphos-methyl and penconazole, respectively. In the case of azinphos-methyl, the exposure remained well below the US EPA guidance value of 560 µg/kg bw and day but would exceed the more conservative CAL EPA reference value in up to 27% of cases when reentry started prior to a 14-day period [36]. Meanwhile, residential exposure to penconazole was determined to be extremely low throughout [34]. The maximum measured value (1.8 µmol/mol creatinine) was 100 times lower than what was excreted in the human dosing study used for establishing the conversion factor. Several of the studies looking at the fungicide exposure of workers in vineyards derived the systemic exposure from the total actual dermal exposure, as the inhalative route is negligible for many of the examined fungicides [25,27]. According to several studies, this also applies to operators for whom inhalative exposure is reported to be as low as 1.1% [39]. The resulting systemic exposure did not exceed the respective reference values in any of the cases investigated. For the cohort of Fustinoni et al. [25], the mean daily exposure was 1.73 (±1.31) µg active substance/kg bw and, as such, clearly below the ADI and AOEL of 0.03 mg/kg bw per day [24,47]. Likewise, in the case of mancozeb, worst-case assumptions resulted in an estimate several hundred times below the AOEL of 0.02 mg/kg bw for the corresponding metabolite (median saturation of 0.01%) [29]. Even the highest individual exposure of workers was still a thousand times below the daily limit, encumbering the reference value of only 1% [31].

Statistically significant correlations between dermal and urinary measurements of metabolites were found in five studies including apples [35] and vine and were observed for operators as well as for workers [24,25,27,29,30,31,32] (Table 3 and Table 4). Reliable knowledge on the correlation of dermal and urinary measurements facilitates the determination of systemic exposure tremendously as it renders the elaborate and costly assessment of dermal patches obsolete, or any other measurements for that matter [25]. Yet, although dermal exposure often constitutes a major route of uptake, other potential routes such as inhalation should always be accounted for. However, the extent to which inhalation contributes to the respective urinary biomarker profile strongly depends on the respective substance and application scenario [35].

A few studies applied exposure models to assess systemic exposure and to predict urinary biomarker concentrations based on information about the type and amount of pesticide applied and culture treated [10,24,26,35]. 

Notably, for most cases, modeling systemic exposure resulted in overestimates when compared to the actual measured values. This applied for the ACROPOLIS model (www.acropolis-eu.com, accessed on 28 February 2022) [24], the UK’s residential exposure assessment (REA) model [26] and the EUROPOEM model [10,26]. With regard to health protection, this suggests that these models were sufficiently conservative.

Predictions of urinary metabolites for captan and penconazole by the REA model, for example, exceeded the actual measured amounts in 98% and 97% of cases. Additionally, for chlorpyrifos and chlormequat, the urinary biomarker concentrations were still higher than predicted, but not significantly different from the background measurements [9].

Similarly, the ACROPOLIS model demonstrated a good predictive performance for the dermal uptake of tebuconazole, albeit in a small cohort (*n* = 7) [24]. Here, the model predicted a mean daily exposure of 1.77 µg active substance/kg bw (±1.96) as opposed to the 1.73 µg active substance/kg bw (±1.31) measured.

Other models such as the AHS pesticide exposure intensity algorithm performed less well [48]. When applied for the assessment of captan in apples and peaches, the algorithm yielded a sufficiently predictive performance for dermal exposure of the thighs and forearms but proved less reliable with regard to overall urinary metabolites or estimates regarding the breathing zone or hand rinse. Hines et al. therefore suggested a revision of the respective exposure weights depending on the method used for application (air blast vs. hand spray) [35].

### 3.5. Health-Related Outcomes

Health-related aspects were explicitly assessed in only one study which looked at the acute effects of mancozeb on the thyroid (Table 3 and Table 4) [28,37]. The study compared two vine-growing areas in Italy, one being iodine-deficient (Chianti), and the other iodine-sufficient (Bolzano). Exposed workers of the first region had lower serum means of free thyroxine (fT4) and showed higher iodine urinary excretion (>250 µg/L ETU as opposed to >20 µg/L ETU). Moreover, workers exposed to mancozeb in the iodine-deficient area tended to have lower thyroid volumes (≤6 mL). These findings fit well with experimental evidence that the thyroid was a major toxicological target of mancozeb as found both in vitro by Lori et al. (2021) and in laboratory animals as summarized by the European Food Safety Authority (EFSA) in a recent evaluation in 2019 [49,50].

## 4. Discussion

This review extends the existing analyses on the exposure of operators, workers, residents and bystanders to pesticides [3,17,18]. It also highlights the need for more systematic studies on this issue. In striking contrast to the numerous claims made in the literature on pesticide exposure, there are surprisingly few studies that looked at this systematically and only in a limited range of target cultures. Focusing on tree-grown produce, vine and hops as typical “overheads” representing a worst case, we found only 11 studies to meet the minimal requirements for any further in-depth analysis. Notably, this was a fraction of the 108 articles initially identified and restricted mainly to apples and vine. Altogether, the respective studies covered a period of 22 years, although the majority were performed in 2010 or thereafter. This helps in so much as one goal of this review was a compilation of the lessons learnt together with an outlook on what could be improved in future studies.

Key issues to consider are the selection of the substance and culture as well as what parameters to (bio)monitor, how to do so and how frequently. One of the lessons learnt in this context is that questionnaires should only serve as means to support the collection of “hard” data, not replace them. Likewise, sole measurement of urinary metabolites without any further data on toxicokinetics or conversion factors will only allow for relative comparisons and thus severely limit any further analysis regarding, for example, the absorbed dose or systemic exposure. This aspect should be considered from the start as both of the latter factors are a precondition for the assessment of any potential health effects, even if only speculative. Conveniently, for some pesticides, the inhalative and oral routes apparently play a negligible role when it comes to total exposure, at least for certain cohorts. In these cases where the dermal route is the main or sole route of exposure, actual skin exposure can be used as a good approximation for systemic exposure.

Sampling of urinary specimens is often the method of choice for its ease of sampling and non-invasive and integrative nature. Yet, the suitability of the matrices selected for monitoring depends on the cohorts, routes of exposure to be assessed, the type of pesticide, the application technique and any potential personal protective equipment used. For the aforementioned groups, the main routes of exposure are usually dermal or inhalative, with the oral route being mainly important in the case of intake of food or ingestion of particles (dust), or as a proxy for inhalation [16]. Therefore, concomitant dermal or inhalative sampling should be considered as appropriate. Both routes are experimentally accessible in a non-invasive manner using pad dosimetry and rinse collection or respiratory filters and air sampling, respectively. Literature searches or experimental pre-screens will also provide data on which routes to consider and by what means [39,40,51]. The detection of particular metabolites shows the occurrence of exposure but fails to inform on the timing, amount or frequency. Without this additional information, further health assessments are not possible. The abundance of pesticide metabolites in urine might hence sufficiently fuel public concern about possible health impacts, but toxicologically, it will inherently remain of limited relevance. State-of-the-art biomonitoring should thus aim for repeated and well-timed sampling to establish levels of systemic exposure, absorbed doses and the respective biokinetics. Only then will it be possible to compare the data to the corresponding reference values and to draw informed conclusions on the likelihood and plausibility of potential adverse health effects.

Depending on the route of uptake, metabolism and the excretion rate, the amount of detectable metabolites can differ widely [6]. This is due to the varying potential influence of the first pass effect. In the case of oral exposure, any xenobiotic will primarily be subjected to hepatic metabolism, but not in cases of dermal or inhalative uptake [16]. This obviously also affects options for back-modeling, for example, when substances with predominantly dermal exposure feature a significant correlation between external measurements and the excreted amount of metabolites.

Kinetic measurements or data pending such cases allow for the calculation of systemic exposure or an absorbed dose merely based on the urinary metabolite concentrations [15,29]. 

Alternatively, one can rely on conversion factors which likewise have to be determined in advance [25,29,30,31].

Finally, biomonitoring constitutes an important pillar in the validation and refinement of toxicokinetic exposure models such as the ACROPOLIS model, the EUROPOEM model or the UK’s REA model. Such models have already shown a high degree of accordance and accuracy in studies where they were used for metabolite and exposure prediction [10,24,26,29,35]. Refined further, they will hence undoubtedly play an increasingly important role in future studies.

## 5. Strengths and Limitations

Modern biomonitoring of pesticide exposure to humans is a topic of high complexity warranting in-depth evaluation of the respective studies and data. This is particularly important when the results are intended to be used for toxicological evaluation or risk assessment. 

The structure and presentation of this scoping review followed the extension of the PRISMA guidelines by Tricco et al. [20]. Comparable methodological approaches for reviews of pesticide exposures were applied in existing reviews [3,17,18]. This includes the use of three different databases and of at least two independent assessments regarding study selection and data extraction [17]. Important features of the respective study design are presented in tabular form, as are the results of the exposure assessment. A further strength of this review is the assessment of the quantification of the respective systemic exposures. Unlike in other reviews which focused on residents only, this review also included groups with potentially higher exposures such as operators and workers [3,17,18].

On the other hand, our review was limited to some overhead cultures where a high degree of exposure to pesticides would be expected. In future, additional overhead cultures such as olive trees should also be considered, in addition to apples, vine and hops. A further limitation of our review might be that it was confined to substances which are currently allowed in Germany and the EU. Although it is true that biomonitoring studies are highly specific to the cultures and substances under investigation, and thus the exclusion of substances which are no longer in use such as DDT might be justified, the inclusion of additional substances would have been certainly of interest, at least from a methodological point of view. 

While sampling of at least urine or serum was a mandatory requirement for the inclusion of articles in this review, analysis of the data clearly highlighted the importance of dermal sampling and inhalative dosimetry. Establishing clear correlations with urinary measurements for both these parameters remains a challenge though, as does establishing clear and unequivocal links to adverse health outcomes. With regard to the latter, one has to differentiate between acute and long-term effects. As shown by the example of mancozeb, it is acute health effects, in particular, that are accessible by biomonitoring if the study is based on a confined cohort and planned accordingly. The situation is more difficult for long-term health effects or chronic endpoints, particularly when relying on large cohort studies such as the Agricultural Health Study (AHS) (https://aghealth.nih.gov, accessed on 28 February 2022). Here, confounding becomes a larger issue, as does exposure monitoring. Long-term exposures can, for example, be monitored by hair measurements, measurement of house dust or environmental parameters such as residues on foliage [3]. However, establishing sufficiently robust dose–response relationships from these data remains scientifically challenging.

## 6. Conclusions

This review is the first to systematically analyze the exposure of operators, workers, residents or bystanders in cultures with potentially high exposure. In these cultures, exposure is predominantly driven by the actual substance application as well as by reentry. As might be expected, this leads to higher exposure for operators and workers than for residents or bystanders. In nearly all cases, comparison of the relevant toxicological reference values to the respective estimated systemic exposure showed the latter to be well below, indicating no reason for concern. This analysis shows the potential of biomonitoring, empowering a hitherto predominantly observational tool to become a toxicologically informative asset for public health protection. As a first step, future studies in this field should therefore aim to systematically establish specific active substance and culture correlations for dermal or inhalative exposure and urinary biomarkers. A framework of established correlations would allow future studies to be based solely on the detection and quantification of metabolites in urine, but performed regularly, such studies could be combined with the monitoring of short- and long-term health effects.

## Data Availability

Not applicable.

## Supplementary Materials

The following supporting information can be downloaded at: https://www.mdpi.com/article/10.3390/toxics10040170/s1, Table S1: Checklist of preferred reporting items for systematic reviews and meta-analyses extension for Scoping Reviews (PRISMA-ScR)—where these items are addressed in the review article by Willenbockel et al. (2022).

## References

[1] Ntzani E.E., Ntritsos G.C.M., Evangelou E., Tzoulaki I. (2013). Literature Review on Epidemiological Studies Linking Exposure to Pesticides and Health Effects.

[2] Ockleford C., Adriaanse P., Berny P., Brock T., Duquesne S., Grilli S., Hougaard S., Klein M., Kuhl T., Laskowski R. (2017). Scientific Opinion of the PPR Panel on the follow-up of the findings of the External Scientific Report ‘Literature review of epidemiological studies linking exposure to pesticides and health effects’. EFSA J..

[3] Dereumeaux C., Fillol C., Quenel P., Denys S. (2020). Pesticide exposures for residents living close to agricultural lands: A review. Environ. Int..

[4] Heudorf U., Butte W., Schulz C., Angerer J. (2006). Reference values for metabolites of pyrethroid and organophosphorous insecticides in urine for human biomonitoring in environmental medicine. Int. J. Hyg. Environ. Health.

[5] Simaremare S.R.S., Hung C.-C., Hsieh C.-J., Yiin L.-M. (2019). Relationship between organophosphate and pyrethroid insecticides in blood and their metabolites in urine: A pilot study. Int. J. Environ. Res. Public Health.

[6] Glorennec P., Serrano T., Fravallo M., Warembourg C., Monfort C., Cordier S., Viel J.F., Le Gléau F., Le Bot B., Chevrier C. (2017). Determinants of children’s exposure to pyrethroid insecticides in western France. Environ. Int..

[7] Roussel C., Witt K.L., Shaw P.B., Connor T.H. (2019). Meta-analysis of chromosomal aberrations as a biomarker of exposure in healthcare workers occupationally exposed to antineoplastic drugs. Mutat. Res. Rev. Mutat. Res..

[8] Sams C., Jones K. (2012). Biological monitoring for exposure to deltamethrin: A human oral dosing study and background levels in the UK general population. Toxicol. Lett..

[9] Eadsforth C.V., Bragt P.C., Van Sittert N.J. (1988). Human dose-excretion studies with pyrethroid insecticides cypermethrin and alphacypermethrin: Relevance for biological monitoring. Xenobiotica.

[10] Galea K.S., MacCalman L., Jones K., Cocker J., Teedon P., Cherrie J.W., van Tongeren M. (2015). Comparison of residents’ pesticide exposure with predictions obtained using the UK regulatory exposure assessment approach. Regul. Toxicol. Pharmacol..

[11] Hays S.M., Aylward L.L., Gagné M., Krishnan K. (2009). Derivation of Biomonitoring Equivalents for cyfluthrin. Regul. Toxicol. Pharmacol..

[12] Quindroit P., Beaudouin R., Brochot C. (2019). Estimating the cumulative human exposures to pyrethroids by combined multi-route PBPK models: Application to the French population. Toxicol. Lett..

[13] Côté J., Bouchard M. (2018). Dose reconstruction in workers exposed to two major pyrethroid pesticides and determination of biological reference values using a toxicokinetic model. J. Expo. Sci. Environ. Epidemiol..

[14] Aylward L.L., Irwin K., St-Amand A., Nong A., Hays S.M. (2018). Screening-level Biomonitoring Equivalents for tiered interpretation of urinary 3-phenoxybenzoic acid (3-PBA) in a risk assessment context. Regul. Toxicol. Pharmacol..

[15] Zoller O., Rhyn P., Zarn J.A., Dudler V. (2020). Urine glyphosate level as a quantitative biomarker of oral exposure. Int. J. Hyg. Environ. Health.

[16] Vermeulen R.C.H., Gooijer I.Y.M., Hoftijser D.G.W., Lageschaar I.L.C.C., Oerlemans D.A., Scheepers D.i.P.T.J., Kivits I.C.M., Duyzer D.J., Gerritsen-Ebben D.M.G., Figueiredo I.D.M. (2019). Research on Exposure of Residents to Pesticides in The Netherlands OBO Flower Bulbs.

[17] Teysseire R., Manangama G., Baldi I., Carles C., Brochard P., Bedos C., Delva F. (2020). Assessment of residential exposures to agricultural pesticides: A scoping review. PLoS ONE.

[18] Teysseire R., Manangama G., Baldi I., Carles C., Brochard P., Bedos C., Delva F. (2021). Determinants of non-dietary exposure to agricultural pesticides in populations living close to fields: A systematic review. Sci. Total Environ..

[19] Galea K.S., MacCalman L., Jones K., Cocker J., Teedon P., Cherrie J.W., van Tongeren M. (2015). Urinary biomarker concentrations of captan, chlormequat, chlorpyrifos and cypermethrin in UK adults and children living near agricultural land. J. Expo. Sci. Environ. Epidemiol..

[20] Tricco A.C., Lillie E., Zarin W., O’Brien K.K., Colquhoun H., Levac D., Moher D., Peters M.D.J., Horsley T., Weeks L. (2018). PRISMA extension for scoping reviews (PRISMA-ScR): Checklist and explanation. Ann. Intern. Med..

[21] Figueiredo D.A.-O., Krop E.A.-O., Duyzer J.A.-O., Gerritsen-Ebben R.A.-O., Gooijer Y.A.-O., Holterman H.A.-O., Huss A.A.-O., Jacobs C.A.-O., Kivits C.A.-O., Kruijne R.A.-O.X. (2021). Pesticide Exposure of residents living close to agricultural fields in The Netherlands: Protocol for an observational study. JMIR Res. Protoc..

[22] Galea K.S., MacCalman L., Jones K., Cocker J., Teedon P., Cherrie J.W., van Tongeren M. (2017). Biological monitoring of pesticides exposure in residents living near agricultural land. Outlooks Pest Manag..

[23] Galea K.S., MacCalman L., Jones K., Cocker J., Teedon P., Sleeuwenhoek A.J., Cherrie J.W., van Tongeren M. (2011). Biological monitoring of pesticide exposures in residents living near agricultural land. BMC Public Health.

[24] Kennedy M.C., Glass C.R., Fustinoni S., Moretto A., Mandic-Rajcevic S., Riso P., Turrini A., van der Voet H., Hetmanski M.T., Fussell R.J. (2015). Testing a cumulative and aggregate exposure model using biomonitoring studies and dietary records for Italian vineyard spray operators. Food. Chem. Toxicol..

[25] Fustinoni S., Mercadante R., Polledri E., Rubino F.M., Mandic-Rajcevic S., Vianello G., Colosio C., Moretto A. (2014). Biological monitoring of exposure to tebuconazole in winegrowers. J. Expo. Sci. Environ. Epidemiol..

[26] Sleeuwenhoek A., Cocker J., Jones K., Cherrie J.W. (2007). Biological monitoring of pesticide exposures. IOM Research Report TM/07/02.

[27] Mercadante R., Polledri E., Rubino F.M., Mandic-Rajcevic S., Vaiani A., Colosio C., Moretto A., Fustinoni S. (2019). Assessment of penconazole exposure in winegrowers using urinary biomarkers. Environ. Res..

[28] Medda E., Santini F., De Angelis S., Franzellin F., Fiumalbi C., Perico A., Gilardi E., Mechi M.T., Marsili A., Citroni A. (2017). Iodine nutritional status and thyroid effects of exposure to ethylenebisdithiocarbamates. Environ. Res..

[29] Mandic-Rajcevic S., Rubino F.M., Ariano E., Cottica D., Negri S., Colosio C. (2019). Exposure duration and absorbed dose assessment in pesticide-exposed agricultural workers: Implications for risk assessment and modeling. Int. J. Hyg. Environ. Health.

[30] Mandic-Rajcevic S., Rubino F.M., Ariano E., Cottica D., Neri S., Colosio C. (2018). Environmental and biological monitoring for the identification of main exposure determinants in vineyard mancozeb applicators. J. Expo. Sci. Environ. Epidemiol..

[31] Mandić-Rajčević S., Rubino F.M., Colosio C. (2020). Establishing health-based biological exposure limits for pesticides: A proof of principle study using mancozeb. Regul. Toxicol. Pharmacol..

[32] Lopez-Galvez N., Wagoner R., Canales R.A., de Zapien J., Calafat A.M., Ospina M., Rosales C., Beamer P. (2020). Evaluating imidacloprid exposure among grape field male workers using biological and environmental assessment tools: An exploratory study. Int. J. Hyg. Environ. Health.

[33] Tao Y., Dong F., Xu J., Phung D., Liu Q., Li R., Liu X., Wu X., He M., Zheng Y. (2019). Characteristics of neonicotinoid imidacloprid in urine following exposure of humans to orchards in China. Environ. Int..

[34] Sams C., Jones K.A.-O., Galea K.S., MacCalman L., Cocker J., Teedon P., Cherrie J.W., van Tongeren M. (2016). Development of a biomarker for Penconazole: A human oral dosing study and a survey of UK residents’ exposure. Toxics.

[35] Hines C.J., Deddens J.A., Jaycox L.B., Andrews R.N., Striley C.A., Alavanja M.C. (2008). Captan exposure and evaluation of a pesticide exposure algorithm among orchard pesticide applicators in the Agricultural Health Study. Ann. Occup. Hyg..

[36] Fenske R.A., Curl C.L., Kissel J.C. (2003). The effect of the 14-day agricultural restricted entry interval on azinphosmethyl exposures in a group of apple thinners in Washington state. Regul. Toxicol. Pharm..

[37] Simcox N.J., Camp J., Kalman D., Stebbins A., Bellamy G., Lee I.-C., Fenske R. (1999). Farmworker exposure to organophosphorus pesticide residues during apple thinning in central Washington State. Am. Ind. Hyg. Assoc. J..

[38] Fustinoni S. Biomonitoring of exposure to penconazole in agriculture. Proceedings of the 31st International Congress on Occupational Health.

[39] Aprea C., Terenzoni B., De Angelis V., Sciarra G., Lunghini L., Borzacchi G., Vasconi D., Fani D., Quercia A., Salvan A. (2004). Evaluation of skin and respiratory doses and urinary excretion of alkylphosphates in workers exposed to dimethoate during treatment of olive trees. Arch. Environ. Contam. Toxicol..

[40] Baldi I., Lebailly P., Jean S., Rougetet L., Dulaurent S., Marquet P. (2006). Pesticide contamination of workers in vineyards in France. J. Expo. Sci. Environ. Epidemiol..

[41] Cao L., Chen B., Zheng L., Wang D., Liu F., Huang Q. (2015). Assessment of potential dermal and inhalation exposure of workers to the insecticide imidacloprid using whole-body dosimetry in China. J. Environ. Sci..

[42] Cao L., Zhang H., Li F., Zhou Z., Wang W., Ma D., Yang L., Zhou P., Huang Q. (2018). Potential dermal and inhalation exposure to imidacloprid and risk assessment among applicators during treatment in cotton field in China. Sci. Total Environ..

[43] Oudbier A.J., Bloomer A.W., Price H.A., Welch R.L. (1974). Respiratory route of pesticide exposure as a potential health hazard. Bull Env. Contam Toxicol.

[44] Hansen J.D., Schneider B.A., Olive B.M., Bates J.J. (1978). Personnel safety and foliage residue in an orchard spray program using azinphosmethyl and captan. Arch. Environ. Contam. Toxicol..

[45] McJllton C.E., Berckman G.E., Deer H.M. (1983). Captan exposure in apple orchards. Am. Ind. Hyg. Assoc. J..

[46] de Cock J., Heederik D., Boleij J.S., Kromhout H., Hoek F., Wegh H., Ny E.T. (1998). Exposure to captan in fruit growing. Am. Ind. Hyg. Assoc. J..

[47] EFSA (2014). Conclusion on the peer review of the pesticide risk assessment of the active substance tebuconazole. EFSA J..

[48] Dosemeci M., Alavanja M.C., Rowland A.S., Mage D., Zahm S.H., Rothman N., Lubin J.H., Hoppin J.A., Sandler D.P., Blair A. (2002). A quantitative approach for estimating exposure to pesticides in the Agricultural Health Study. Ann. Occup. Hyg..

[49] Lori G., Tassinari R., Narciso L., Udroiu I., Sgura A., Maranghi F., Tait S. (2021). Toxicological comparison of mancozeb and zoxamide fungicides at environmentally relevant concentrations by an in vitro approach. Int. J Environ. Res. Public Health.

[50] Abdourahime H., Anastassiadou M., Arena M., Auteri D., Barmaz S., Brancato A., Bura L., Cabrera L.C., Chaideftou E., European Food Safety Authority (EFSA) (2019). Conclusion on the peer review of the pesticide risk assessment of the active substance mancozeb. EFSA J..

[51] Baldi I., Lebailly P., Bouvier G., Rondeau V., Kientz-Bouchart V., Canal-Raffin M., Garrigou A. (2014). Levels and determinants of pesticide exposure in re-entry workers in vineyards: Results of the PESTEXPO study. Environ. Res..

